# Efficacy and Safety of Insulin Degludec/Insulin Aspart Compared with a Conventional Premixed Insulin or Basal Insulin: A Meta-Analysis

**DOI:** 10.3390/metabo11090639

**Published:** 2021-09-18

**Authors:** Shinje Moon, Hye-Soo Chung, Yoon-Jung Kim, Jae-Myung Yu, Woo-Ju Jeong, Jiwon Park, Chang-Myung Oh

**Affiliations:** 1 Department of Internal Medicine, College of Medicine, Hallym University, Chuncheon 24252, Korea; sinjei1129@gmail.com (S.M.); soo3802@hanmail.net (H.-S.C.); eun99star@naver.com (Y.-J.K.); jaemyungyu@hallym.or.kr (J.-M.Y.); 2 Department of Biomedical Science and Engineering, Gwangju Institute of Science and Technology, Gwangju 61005, Korea; uju00112@gm.gist.ac.kr (W.-J.J.); jiwon3803@gm.gist.ac.kr (J.P.)

**Keywords:** type 2 diabetes, insulin degludec, insulin aspart, premixed insulin, glycemic control, hypoglycemia

## Abstract

Insulin degludec/insulin aspart (IDegAsp) is a novel co-formulation of 70% insulin degludec and 30% insulin aspart. The present meta-analysis was conducted to assess the efficacy and safety of IDegAsp compared with a conventional premixed insulin or basal insulin. We extracted data from citation databases, including PubMed, EMBASE, and the Cochrane Library, since inception to 2021. We calculated the mean differences for hemoglobin A1c (HbA1c), fasting plasma glucose (FPG), self-measured mean glucose, and postprandial glucose (PPG) and odds ratios for confirmed hypoglycemia events. Compared with twice-daily conventional premixed insulin, twice-daily IDegAsp showed a similar effect on changes in HbA1c, but it significantly reduced FPG and self-measured mean glucose levels. Furthermore, compared to once-daily basal insulin, once-daily IDegAsp had a similar effect on changes in HbA1c, but it significantly reduced self-measured mean glucose and PPG levels. The risk of overall confirmed hypoglycemia was similar between treatments; however, the risk of nocturnal hypoglycemia events was significantly lower with IDegAsp than with conventional premixed insulin and basal insulin. Thus, IDegAsp was more effective than conventional premixed insulin and basal insulin at reducing blood glucose with fewer nocturnal hypoglycemia events.

## 1. Introduction

For people with type 2 diabetes (T2D) who cannot achieve optimal blood glucose levels with basal insulin and oral glucose-lowering agents, intensive treatment regimens, such as basal insulin with mealtime rapid-acting insulin regimen (basal-bolus insulin therapy) or premixed insulin regimens are imperative [1].

Because of the development of basal insulin, such as insulin glargine (IGlr; Lantus^®^, a long-acting insulin analogue) and insulin degludec (IDeg, Tresiba^®^, an ultra-long-acting insulin analogue), the basal-bolus insulin regimen is generally used for glucose control because it more closely mimics the physiologic insulin secretion pattern compared to the premixed insulin regimen [2]. However, the premixed insulin regimen is also widely used as a well-established regimen for glucose control [3]. The biphasic insulin aspart (BIAsp30; NovoMix^®^ 30), containing 30% soluble insulin aspart and 70% protamine-crystallized insulin aspart, and biphasic insulin lispro (lispro mix25; Humalog^®^ Mix25), containing 25% soluble insulin lispro and 75% protamine-crystallized insulin lispro, are the most commonly used premixed insulins [4]. Premixed insulin can cover postprandial glucose control, which the basal insulin-only regimen cannot cover, and this regimen a more accessible form of T2D management because it requires only two or three shots a day compared to the basal-bolus insulin regimen, which requires four to five shots a day [4].

Insulin degludec/insulin aspart (IDegAsp; Ryzodeg^®^) is a novel co-formulation of 70% IDeg and 30% insulin aspart (IAsp, a rapid-acting insulin) administered as a single injection, either once or twice daily with main meals [2], wherein each insulin exerts its glucose-lowering effects without establishing interactions with the other agents [5,6].

After IDegAsp was approved by the European Medicines Agency (EMA) in 2013 and the US Food and Drug Administration (FDA) in 2015, this drug was widely used globally for achieving robust glycemic control with low risk of hypoglycemia development [7]. IDegAsp provides a prolonged and steady basal glucose-lowering effect attributable to the IDeg component and reduces post-meal glucose spikes, owing to the IAsp component [7].

The clinical effects of this new combination have been reported in several clinical trials [8,9,10]. The IDegAsp co-formulation has shown favorable benefits compared with other current insulin regimens, such as basal-only, premixed insulin and basal-bolus regimens [6]. However, there is a lack of consensus regarding switching from conventional insulin regimens to this new IDegAsp regimen because few trials have recently reported the non-inferiority of IDegAsp compared with other insulins [11,12].

The aim of the present meta-analysis is to compare the efficacy and safety of IDegAsp co-formulation compared with a conventional premixed insulin, such as biphasic insulin aspart 30 (BiAsp30), or basal insulin, such as insulin glargine or IDeg. To evaluate the benefits of the IDegAsp regimen, we analyzed the blood glucose level and insulin dose of the study subjects. We also analyzed hypoglycemic events to evaluate safety.

## 2. Results

### 2.1. Study Characteristics

**Figure 1:**
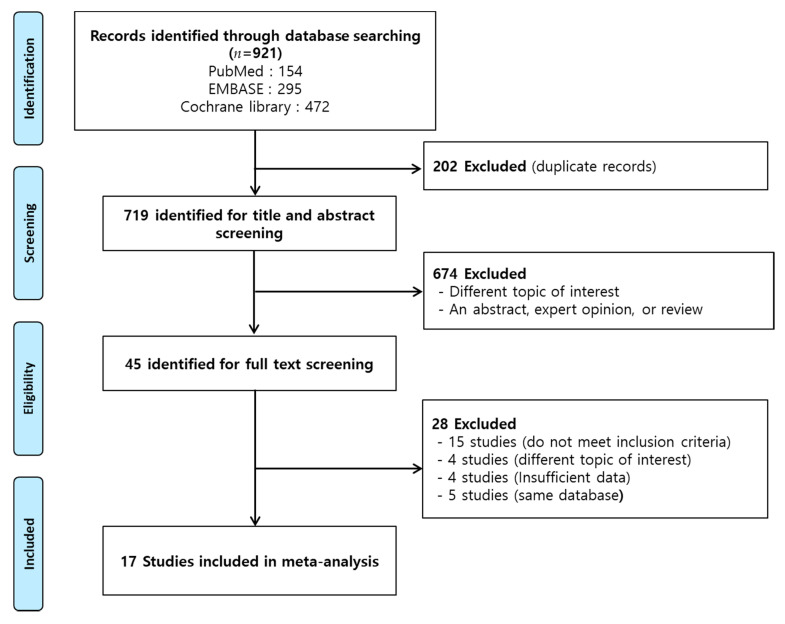
Schema of the search strategy.

The literature search yielded 921 potentially relevant articles (PubMed 154, EMBASE 295, and Cochrane Library 472), of which 719 were screened for title and abstract after excluding 202 duplicate articles. Thereafter, 674 articles that did not meet the inclusion criteria were excluded, and the remaining 45 studies were evaluated for eligibility by performing a full-text review; finally, 17 studies [10,13,14,15,16,17,18,19,20,21,22,23,24,25,26,27,28] with 3831 participants were included in this meta-analysis (Figure 1). The effect on glycemic control and the risk of hypoglycemia development with those on the twice-daily IDegAsp regimen were reported in eight studies [13,14,15,16,17,18,19,20], while those on the once-daily regimen were reported in nine studies [10,21,22,23,24,25,26,27,28]. The demographic characteristics and main outcomes of each study are summarized in Table 1. Of the 17 studies, 13 were randomized controlled trials (RCTs) and reported low risk of bias [10,15,16,17,19,20,22,24,25,26,27,28]. We classified one RCT with a serious risk of bias as we used the data to only determine the switch from once-daily basal insulin to once-daily IDegAsp [23]. Three trials with a one-group pretest–post-test design demonstrated a serious risk of bias [13,14,21].

### 2.2. The Effect of Twice-Daily IDegAsp on Glycemic Control Compared to Conventional Premixed Insulin

**Figure 2:**
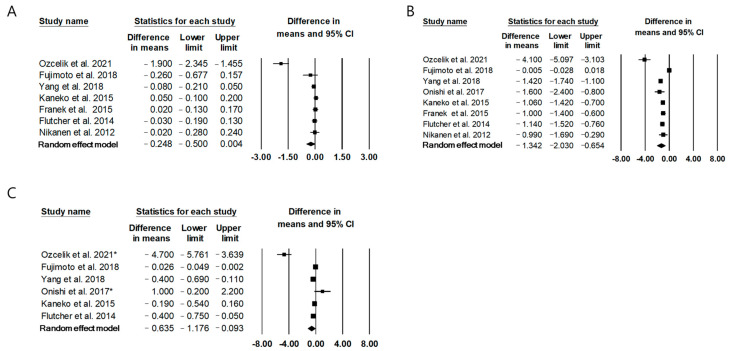
Forest plots summarizing the effect of IDegAsp on glycemic control compared to a conventional premixed insulin. (**A**) HbA1C, (**B**) FPG (**C**) Mean self-measured glucose level. * Mean self-measured PPG.

Eight studies [13,14,15,16,17,18,19,20] comprising 2085 patients reported the effect of twice-daily IDegAsp on glycemic control compared to that of twice-daily conventional premixed insulin. The mean difference (MD) in hemoglobin A1C (HbA1c) between IDegAsp and conventional premixed insulin was −0.25 (95% confidence interval (CI) −0.50 to 0.00), which indicated a similar effect on the change in HbA1c with both treatments, and the I^2^ was 91.4%, indicating significant heterogeneity (Figure 2A). The publication bias was not detected (Egger’s test: *p* = 0.09) [10,11,12,14,15,16,17]. Subgroup analysis with five RCTs [15,17,18,19,20] showed a similar result (MD −0.02; 95% CI −0.09 to 0.06).

IDegAsp significantly reduced fasting plasma glucose (FPG) compared with conventional premixed insulin (MD −1.34 mmol/L, 95% CI −2.03 to −0.65, I^2^ = 97.1%; Figure 2B) [13,14,15,16,17,18,19,20]. Significant publication bias was found (Egger’s test: *p* < 0.01); therefore, the trim-and-fill method was used to adjust the bias by adding two estimated missing studies. The statistical significance was maintained after publication bias adjustment (MD −1.64 mmol/L, 95% CI −2.99 to −0.29; Figure S1A). The sensitivity analysis showed that the significance of the MD did not change even after each study was omitted (Figure S2A). Subgroup analysis with six RCTs [15,16,17,18,19,20] showed a significant reduction in FPG in the IDegAsp group than in the conventional premixed insulin group (MD −1.19 mmol/L, 95% CI −1.36 to −1.02, I^2^ = 0%).

IDegAsp significantly reduced mean self-measured glucose levels compared with conventional premixed insulin (MD −0.64 mmol/L, 95% CI −1.18 to −0.09, I^2^ = 94.4%; Figure 2C) [13,14,15,16,17,19]. The publication bias was not detected (Egger’s test: *p* = 0.21; Figure S1B). Moreover, statistical significance could not be observed in the sensitivity analysis after an outlier study [13] was removed (Figure S2B). Subgroup analysis with four RCTs [15,16,17,19] showed a significant reduction in self-measured mean glucose levels in the IDegAsp group compared to the conventional premixed insulin group (MD –0.31 mmol/L, 95% CI −0.49 to −0.12, I^2^ = 46.9%).

### 2.3. The Effect of Once-Daily IDegAsp on Glycemic Control Compared to Basal Insulin

**Figure 3:**
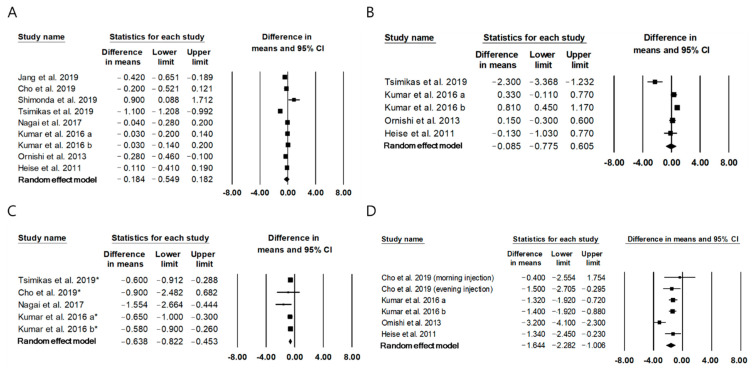
Forest plots summarizing the effect of IDegAsp on glycemic control compared to a basal insulin. (**A**) HbA1C, (**B**) FPG, (**C**) mean self-measured glucose level, (**D**) PPG. * Mean self-measured PPG.

Nine studies [10,21,22,23,24,25,26,27,28] comprising 1636 patients reported the effect of once-daily IDegAsp on glycemic control compared to that of once-daily basal insulin. The MD in HbA1c between the IDegAsp and basal insulin groups was −0.18 (95% CI, −0.55 to 0.18), indicating a similar effect on the change in HbA1c with both treatments; the I^2^ was 94.9%, indicating significant heterogeneity (Figure 3A). The publication bias was not detected (Egger’s test: *p* = 0.06). Subgroup analysis with seven RCTs [10,22,24,25,26,27,28] showed a similar result (MD, −0.07; 95% CI, −0.21 to 0.06).

Change in FPG levels was similar for IDegAsp versus basal insulin (MD −0.09 mmol/L, 95% CI −0.78 to 0.61, I^2^ = 87.4%; Figure 3B). A significant publication bias was not detected (Egger’s test, *p* = 0.06) [23,25,26,27,28]. Furthermore, subgroup analysis with four RCTs [25,26,27,28] did not show a significant difference in FPG between the IDegAsp and basal insulin groups.

IDegAsp significantly reduced mean self-measured glucose level (MD −0.64 mmol/L, 95% CI −0.82 to −0.45; Figure 3C) compared to basal insulin, without significant heterogeneity (I^2^ = 0%) [22,23,24,25]. The funnel plot was symmetrical, and publication bias was not detected (Egger’s test: *p* = 0.11; Figure S1C). In the sensitivity analysis, the significance of the results did not change even after each study was removed (Figure S2C). Subgroup analysis with four RCTs [22,24,25,26] showed a similar significant result (MD −0.65 mmol/L, 95% CI −0.88 to −0.42, I^2^ = 26.8%).

Five RCTs [22,23,24,25,26] reported that IDegAsp significantly reduced postprandial glucose (PPG) after insulin injection compared to basal insulin (MD −1.64 mmol/L, 95% CI −2.28 to −1.01, I^2^ = 66.6%; Figure 3D). The funnel plot was symmetrical, and publication bias was not detected (Egger’s test: *p* = 0.91; Figure S1D). In the sensitivity analysis, the significance of the results did not change even after each study was removed (Figure S2D).

### 2.4. Risk of Hypoglycemia with IDegAsp

**Figure 4:**
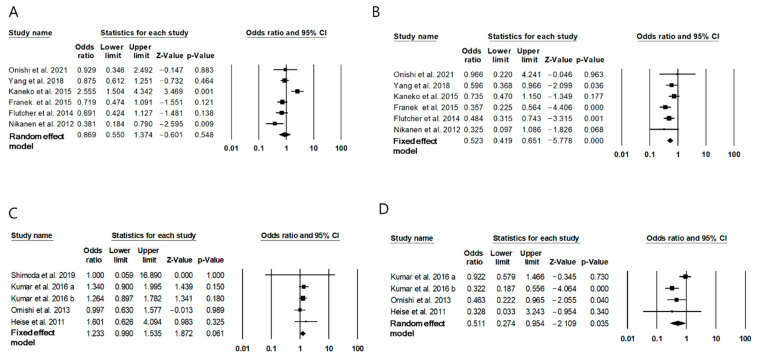
Odds ratios for hypoglycemia of IDegAsp. (**A**) OR for the overall confirmed hypoglycemia in IDegAsp compared to a conventional premixed insulin, (**B**) OR for the nocturnal confirmed hypoglycemia in IDegAsp compared to a conventional premixed insulin, (**C**) OR for the overall confirmed hypoglycemia in IDegAsp compared to basal insulin, (**D**) OR for the nocturnal confirmed hypoglycemia in IDegAsp compared to basal insulin.

At least one overall confirmed case of hypoglycemia was reported in 728 of 1129 (64.5%) participants who were administered with twice-daily IDegAsp in six RCTs [15,16,17,18,19,20], and 541 of 1269 (67.6%) participants who were administered with conventional premixed insulin. The risk of overall confirmed hypoglycemia in the twice-daily IDegAsp group was not significantly different from that in the twice-daily B30 group (odds ratio (OR) 0.87, 95% CI 0.55 to 1.37, I^2^ = 77.7%; Figure 4A). Furthermore, 212 of 1129 (18.8%) participants who were administered with twice-daily IDegAsp and 463 of 1929 (31.4%) participants who were administered with conventional premixed insulin experienced at least one nocturnal hypoglycemia event. Thus, the risk of nocturnal confirmed hypoglycemia was significantly lower in the twice-daily IDegAsp group (OR 0.52, 95% CI 0.42 to 0.65, I^2^ = 23.9%; Figure 4B) than that in the twice-daily conventional premixed insulin group. A publication bias was not detected (Egger’s test: *p* = 0.94; Figure S1E). The sensitivity analysis showed that the significance of the OR did not change even after each study was omitted (Figure S2E).

Among 694 participants administered with once-daily IDegAsp in five RCTs [10,25,26,27,28] and 703 participants administered with once-daily basal insulin, 353 (50.9%) and 324 (46.1%) participants had at least one overall confirmed hypoglycemia event, respectively. The risk of overall confirmed hypoglycemia in the once-daily IDegAsp group was similar to that in the once-daily basal insulin group (OR 1.23, 95% CI 0.99 to 1.54, I^2^ = 0%; Figure 4C). Furthermore, among 668 participants administered with once-daily IDegAsp in four studies [25,26,27,28] and 677 participants administered with once-daily basal insulin, 77 (11.5%) and 129 (19.1%) demonstrated at least one nocturnal confirmed hypoglycemia event, respectively. Thus, the risk of nocturnal confirmed hypoglycemia was significantly lower in the once-daily IDegAsp group (OR 0.51, 95% CI 0.27 to 0.95, I^2^ = 66.0; Figure 4D) than that in the once-daily basal insulin group. Publication bias was not detected (Egger’s test: *p* = 0.64).

## 3. Discussion

IDegAsp is the first soluble combination of 70% ultra-long-acting insulin (IDeg) and 30% rapid-acting insulin (IAsp) [29]. IDeg is a basal insulin analog with a half-life of over 25 h and can exert a stable glucose-lowering effect [30]. IAsp is a pre-meal insulin analog with a faster onset (10–15 min) and shorter duration (4–5 h) of action than regular human insulin [31]. This soluble combination of two individual insulin analogs can be administered once or twice daily to people with type 2 diabetes and has demonstrated its safety and efficacy in clinical studies [18,30,32].

In the meta-analysis involving eight studies conducted on the comparison of twice-daily IDegAsp with twice-daily conventional premixed insulin [13,14,15,16,17,18,19,20], it was found that both regimens significantly reduced HbA1c levels, but the difference between both groups was not significant. However, twice-daily IDegAsp significantly reduced FPG and mean self-measured glucose levels more effectively than twice-daily conventional premixed insulin. The indicated glucose-lowering effects, as observed with FPG and mean glucose levels, are mainly attributed to the impact of the long-acting insulin analog (IDeg) in the IDegAsp regimen. Previous studies reported the superiority of the twice-daily long-acting insulin regimen for glucose control compared with other regimens [11,33]. For example, twice-daily insulin glargine improved glycemic control in patients with uncontrolled diabetes after switching from a once-daily regimen to a twice-daily regimen [33]. Furthermore, twice-daily basal insulin treatment showed better glycemic control with a lower risk of hypoglycemia than both twice-daily neutral protamine Hagedorn (NPH) and once-daily basal insulin treatments [34].

Compared with once-daily basal insulin [10,21,22,23,24,25,26,27,28], once-daily IDegAsp resulted in a similar reduction in HbA1c and FPG levels. However, once-daily IDegAsp significantly reduced mean self-measured glucose level and PPG more effectively than once-daily basal insulin. The IAsp component of IDegAsp helps achieve meal-time glycemic control, and this explains the beneficial effects observed in average blood glucose (HbA1c and self-measured mean glucose level) and PPG levels. However, in general, FPG is controlled by the action of long-acting basal insulin. Thus, IDegAsp was non-inferior to basal insulin in controlling FPG.

Intriguingly, the total insulin dose required in the IDegAsp group was lower than [23,28] or similar to that in the basal insulin group [10,21,22,24,26,27]. Furthermore, only one study among nine studies [25] showed that more insulin was necessary for the IDegAsp group than the basal insulin group to control their blood glucose levels. This implies that IDegAsp is a better once-daily insulin regimen than other basal insulin regimens for people with type 2 diabetes for insulin dose control as well as glucose control.

Regarding safety, we investigated the event rates of overall hypoglycemia and nocturnal hypoglycemia with IDegAsp and other insulin therapies. IDegAsp was more effective than conventional premixed insulin and basal insulin in reducing blood glucose levels with the occurrence of fewer nocturnal hypoglycemia events. Compared with a conventional premixed insulin, twice-daily IDegAsp showed similar overall hypoglycemia risk and significantly lower risk of nocturnal hypoglycemia. This reflects the safety profile of long-acting IDeg, which provides continuous, slow, and stable basal insulin coverage, thereby reducing the risk of hypoglycemia [11]. On the contrary, an intermediate action NPH, a basal component of premixed insulin, shows peak activity 5–8 h after injection and demonstrates a high risk for hypoglycemia [11]. Furthermore, a recent meta-analysis, including 24 RCTs, reported that these long-acting insulin analogs exhibited a reduced risk of severe hypoglycemia compared to NPH (insulin glargine versus NPH: relative risk 0.68, 95% CI 0.46 to 1.01; insulin detemir versus NPH: relative risk 0.45, 95% CI 0.17 to 1.20) [11].

Compared with once-daily basal insulin, once-daily IDegAsp showed similar overall hypoglycemia risk and significantly lower risk of nocturnal hypoglycemia. This might be attributable to the absence of peak activity and pharmacodynamic effects extending over 24 h of IDeg administration. In addition, IDeg demonstrates a longer duration of action and less variability compared to other basal insulin analogs (insulin glargine and insulin detemir) [35]. Thus, real-world data have reported that IDeg exerts more effective glucose-lowering effects and demonstrates lower hypoglycemic risk compared to other basal insulin analogs [11,36].

This is the first meta-analysis to evaluate the effect of IDegAsp on glycemic control without switching insulin regimens. However, this study has some limitations. First, we could not conduct various subgroup analyses according to age, HbA1c, and underlying metabolic diseases due to the small number of studies. Second, we could not analyze the beneficial effect of switching from conventional insulin to IDegAsp in terms of preventing and improving diabetic complications. Further long-term follow-up clinical trials are needed to overcome these limitations.

## 4. Materials and Methods

### 4.1. Search Strategy

The literature search was conducted according to the Preferred Reporting Items for Systematic Reviews and Meta-Analyses (PRISMA) guidelines (Table S1). Moon S. extracted data from citation databases (PubMed, EMBASE, and the Cochrane Library) using search terms that included combinations of “degludec” and “aspart” from the inception of the database to 2021. Oh C m verified the data for accuracy (Table S2).

### 4.2. Study Selection

The inclusion criteria were as follows:

1. Population: patients with diabetes mellitus (DM) who used insulin injection.
2. Intervention: once- or twice-daily injection of IDegAsp for 4 weeks or more.
3. Comparators: patients with a conventional premixed insulin or basal insulin in RCTs or non-RCTs, or patients using a conventional premixed insulin or basal insulin before IDegAsp intervention in studies with a one-group pretest–posttest design.
4. Outcomes: data on changes in HbA1C, FPG, self-measured mean glucose, or PPG and number of confirmed hypoglycemia events.
5. Study design: clinical trials using IDegAsp.

The exclusion criteria were as follows:

1. In vivo or in vitro experiments, only abstracts, and non-original articles, including expert opinions or reviews.
2. Studies on type 1 DM.
3. Studies involving participants who used multiple-dose insulin injection therapy or glucagon-like peptide-1 (GLP-1) agonist.
4. Studies involving participants with diseases, such as cancer, that affected the outcomes.

### 4.3. Data Extraction

The following variables were extracted from the eligible studies: first author, publication year, characteristics of the participants, number of study participants according to intervention, mean age, FPG, PPG, HbA1c, and number of confirmed hypoglycemia events.

### 4.4. Quality Assessment

The quality of studies was evaluated by two researchers using the Revised Cochrane risk-of-bias tool for randomized trials (RoB 2.0). Studies based on a one-group pretest–post-test design are considered to exhibit a critical risk of bias. Discrepancies in quality assessment were resolved through discussions with a third investigator (Yu J.M.).

### 4.5. Statistical Methods

The pooled effect sizes of continuous variables were presented as MD and 95% CIs between the IDegAsp intervention group and the control group with a conventional premixed insulin or basal insulin therapy. Additionally, we calculated the pooled ORs with 95% CIs for dichotomous variables using the Mantel–Haenszel method. The Cochrane Q statistic and Higgins I^2^ statistic were used to determine heterogeneity. In the present study, we considered that I^2^ ≤ 50% indicated less remarkable heterogeneity between the studies and used a fixed-effects model to estimate the pooled effect sizes; in contrast, we considered that I^2^ > 50% indicated significant heterogeneity and used a random-effects model therein. The publication bias was tested using the Egger test and a funnel plot. Sensitivity and subgroup analyses were used to determine the cause of heterogeneity. The Comprehensive Meta-Analysis software version 3 (Biostat, Englewood, NJ, USA) was used for this meta-analysis.

## 5. Conclusions

In conclusion, findings of this meta-analysis show that both once-daily and twice-daily IDegAsp regimens demonstrate efficacy and safety benefits compared to other insulin regimens. Furthermore, switching from premixed insulin and basal insulin to IDegAsp does not increase the patient’s burden and positively impacts glycemic control. Further long-term large-scale follow-up clinical trials are needed to overcome these limitations and provide the metabolic benefits and safety of IDegAsp regimens.

## Figures and Tables

**Table 1:** Summary of the 17 studies included in the present meta-analysis.

Study [Ref]	Study Design	Characteristics of Participants	Study Duration	No. of Participants	Age (Years), Mean ± SD or Median (IQR)	Main Outcomes, Mean Difference ± SD or Mean Difference (95%CI)	Insulin Type and Dose, Mean ± SD or Median (IQR)	Reports of Hypoglycemia, Events or Estimated Treatment Ratio (%)
Clinical trials comparing IGegAsp with the conventional premixed insulin (eight studies)								
Ozcelik et al., 2021 [13]	Premixed insulin for at least 3 months, then replaced insulin by IDegAsp for 12 weeks	Type 2 diabetes, Age ≥ 18, Patients on premixed in addition to oral antidiabetic drugs	12 weeks	55	67.0 (62–69) *	FPG: −72.9 ± 79.3 mg/dL PPG: −84.8 ± 86.6 mg/dL HbA1c: −1.90 ± 1.96%	I: IDegAsp; 38(36–40) U/day C: BIAsp30 or insulin lispro 25/75; 48 (40–55) U/day	Overall (event/week) I: 0.03 ± 0.11 C: 1.5 ± 0.85
Fujimoto et al., 2018 [14]	Premixed insulin for 2 months, then followed by IDegAsp for the next 2 months	Type 2 diabetes, Patients on premixed insulin therapy twice daily for more than 6 months.	4 months	22	68.0 ± 9.9	MBG: −0.46 (−1.05, 0.14) mg/dL HbA1c: −0.26 (−0.85, 0.33)%	I: IDegAsp; Morning 8.05 ± 0.78 U/day, Evening 7.77 ± 0.72 U/day C: BIAsp or biphasic human insulin; Morning 7.91 ± 0.70 U/day, Evening 8.23 ± 0.76	0 event per month in both group
Yang et al., 2019 [15]	RCT I: twice daily IDegAsp C: twice daily BIAsp30	Type 2 diabetes, Age ≥ 18, Patients on once- or twice- daily premixed insulin or basal insulin ± metformin	26 weeks	I:360 C:181	I: 59.6 ± 9.0 C: 58.8 ± 9.4	FPG: −1.42 (−1.74, −1.10) mmol/L HbA1c: non-inferior	I: IDegAsp; 0.78 ± 0.35 U/kg/day C: BIAsp30; 0.95 ± 0.35 U/kg/day	Nocturnal: 47% Total: 43%
Onishi et al., 2017 [16]	RCT I: twice daily IDegAsp C: twice daily BIAsp30	Type 2 diabetes, Age ≥ 20, Patients on twice-daily on same basal insulin or premixed insulin	6 weeks	I: 33 C: 32	I: 64.3 ± 8.4 C: 64.7 ± 11.2	FPG: −1.6 (−2.4, −0.8) mmol/L PPG: 1.0 (−0.1, 2.2) mmol/L Mean PG: −0.4 (−1.3, 0.5) mmol/L	I: IDegAsp; Morning 11.4–12.7 U/day, Evening 10.5–10.7 U/day C: BIAsp30; Morning 12.3–13.3 U/day, Evening 9.9–11.8 U/day	Nocturnal: 51% Total: 47%
Kaneko et al., 2015 [17]	RCT I: twice daily IDegAsp C: twice daily BIAsp30	Type 2 diabetes, Age ≥ 18 or 20, Patients on once- or twice- daily premixed insulin or basal insulin ± metformin	26 weeks	I: 282 C: 142	I: 59.1 ± 10.2 C: 61.2 ± 9.5	FPG: −1.06 (−1.43, −0.70) mmol/L Mean PG: −0.19 (−0.54, 0.16) HbA1c: 0.05 (−0.10, 0.20)%	I: IDegAsp; Morning 34 U/day, Evening 21 U/day C: BIAsp30; Morning 38 U/day, Evening 30 U/day	Nocturnal: NS Total: NS
Franek et al., 2015 [18]	RCT I: twice daily IDegAsp C: twice daily BIAsp30	Type 2 diabetes, Age ≥ 18, insulin naïve patients	26 weeks	I: 197 C: 197	I: 59.0 ± 9.5 C: 58.8 ± 8.4	FPG: −1.0 (−1.4, −0.6) mmol/L Mean PG: NS HbA1c: 0.02 (−0.12, 0.17)%	I: IDegAsp; Morning 0.44 U/kg/day, Evening 0.35 U/kg/day C: BIAsp30; Morning 0.42 U/kg/day, Evening 0.40 kgU/day	Nocturnal: 73% Total: 32%
Flutcher et al., 2014 [19]	RCT I: twice daily IDegAsp C: twice daily BIAsp30	Type 2 diabetes, Age ≥ 18 or 20, Patients on once- or twice- daily premixed insulin ± oral hypoglycemic agents	26 weeks	I: 224 C: 223	I: 58.7 ± 9.9 C: 58.8 ± 9.8	FPG: −1.14 (−1.53, −0.76) mmol/L Mean PG: −0.4 (−0.75, −0.05) mmol/L HbA1c: −0.03 (−0.18, 0.13)%	I: IDegAsp; Morning 38 U/day, Evening 52 U/day C: BIAsp30; Morning 44 U/day, Evening 54 U/day	Nocturnal: 74% Total: 54%
Nikanen et al., 2012 [20]	RCT I: twice daily IDegAsp C: twice daily BIAsp30	Type 2 diabetes, 75 ≥ Age ≥ 18, insulin naïve patients or patients with insulin treatment within 14 days in the 3 months prior to trial	16 weeks	I: 61 C: 62	I: 58.7 ± 8.5 C: 59.7 ± 8.0	FPG: −0.99 (−1.68, −0.29) mmol/L Mean PG: NS HbA1c: −0.02 (−0.27, 0.24)%	I: IDegAsp; 0.57 ± 0.23 U/kg/day C: BIAsp30; 0.66 ± 0.30 U/kg/day	Nocturnal: 74% Total: 67%
Clinical trials comparing IDegAsp with Basal insulin (nine studies)								
Jang et al., 2019 [21]	Basal insulin for at least 4 months, then replaced insulin by IDegAsp	Type 2 diabetes	3 months basal insulin, then 3 months IDegAsp	80	67 ± 9.8	HbA1c: −0.4%	I: IDegAsp; 0.39 ± 015 U/kg/day C: IGlr-100 or IGlr-300 or insulin detemir or IDeg; 0.36 ± 0.14 U/kg/day	NR
Cho et al., 2020 [22]	RCT I: once daily IDegAsp C: once daily basal insulin (IDeg or IGlr)	Type 2 diabetes, 80 ≥ Age ≥ 20	12 weeks	I: 30 C: 29	I: 64.8 ± 1.8 C: 63.3 ± 1.9	FPG: NS PPG: −0.9 mmol/L HbA1c: NS	I: IDegAsp; 13.7 ± 8.9 U/day C: IGlr; 13.7 ± 6.9 U/day	NR
Shimonda et al., 2019 [10]	RCT I: once daily IDegAsp C: once daily basal insulin (IDeg or IGla-300)	Type 2 diabetes, Age ≥ 20	12 weeks	I: 26 C: 26	I: 57.0 (48.0–68.0) C: 49.0 (45.0–60.0)	HbA1c: NS	I: IDegAsp; 0.154 (0.143–0.198) U/kg/day C: IDeg; 0.145 (0.128–0.158), IGlr-300; 0.189 (0.160–0.220) U/kg/day	NR
Tsimikas et al., 2019 [23]	RCT I: once daily IDegAsp C: once daily basal insulin (IGla-100) + IAsp)	Type 2 diabetes, Age ≥ 18	26 weeks	I: 267 C: 265	I: 58.2 ± 8.9 C: 59.2 ± 9.1	FPG: −2.3 ±2.9 mmol/L HbA1c: −1.1 ± 0.9% Mean PG: −0.6 ± 2.6 mmol/L	I: IDegAsp; 83.4 ± 51.3 U/day C: IGlr-100; 89.3 ± 43.1 U/day	Nocturnal (events/100 person-years of exposure) I: 47.9, C:92.9 Overall (events/100 person-years of exposure) I: 258.5, C: 296.1
Nagai et al., 2017 [24]	RCT I: once daily IDegAsp C: once daily basal insulin	Type 2 diabetes, Age ≥ 20 or 20, Patients on once-daily basal insulin plus oral hypoglycemic agents	4 weeks	I: 12 C: 11	I: 66 ± 13 C: 68 ± 8	Mean PG: −28 (−47, −8) mg/dL HbA1c: NS	I: IDegAsp; 16.9 ± 6.8 U/day C: IGlr or IDeg; 23.0 ± 7.8 U/day	NS
Kumar et al., 2016 [25]	RCT I: once daily IDegAsp C: once daily basal insulin (IGla)	Type 2 diabetes, Age ≥ 18, insulin naïve patients	52 weeks	I: 266 C: 263	I: 57.4 ± 9.0 C: 56.4 ± 9.2	FPG: NS PPG: −0.34 (−0.64, −0.04) mmol/L HbA1c: −0.08 (−0.26, 0.09)%	I: IDegAsp; 66 U/day C: IGlr-100; 59 U/day	Nocturnal: 75% Total: 186%
Kumar et al., 2017 [26]	RCT I: once daily IDegAsp C: once daily basal insulin (IGlar)	Type 2 diabetes, Age ≥ 18, Patients on once-daily basal insulin plus oral hypoglycemic agents	26 weeks	I: 196 C: 205	I: 57.8 ± 9.5 C: 58.4 ± 10.1	FPG: NS Mean PG: 0.55 (0.23, 0.88) mmol/L, HbA1c: −0.03 (−0.20, 0.14)%	I: IDegAsp; 60 U/day C: IGlr; 60 U/day	NS
Onishi et al., 2013 [27]	RCTI : once daily IDegAsp C: once daily basal insulin (IGlar)	Type 2 diabetes, Age ≥ 20, insulin naïve patients	26 weeks	I: 147 C: 149	I: 60.0 ± 10.0 C: 61.0 ± 9.6	FPG: NS Mean PG: −3.2 (−4.1, −2.3) mmol/L HbA1c: −0.28 (−0.46, −0.10)%	I: IDegAsp; 28 U/day C: IGlr-100; 29 U/day	NS
Heise et al., 2011 [28]	RCT I: once daily IDegAsp C: once daily basal insulin (IGlar)	Type 2 diabetes, 75 ≥ Age ≥ 18, insulin naïve patients	16 weeks	I: 100 C: 100	I: 58.7 ± 8.8 C: 58.4 ± 8.4	FPG: −0.13 (−1.03, 0.77) mmol/L Mean PG: −1.34 (−2.45, −0.23) mmol/L HbA1c: −0.11 (−0.41, 0.19)	I: IDegAsp; 0.38 ± 0.16 U/kg/day C: IGlr-100; 0.45 ± 0.20 U/kg/day	NS

FPG, fasting plasma glucose; PPG, postprandial plasma glucose; HbA1c, hemoglobulin A1C; MBG, mean blood glucose; PG, plasma glucose; RCT, randomized controlled trial; BIAsp30, biphasic insulin aspart 30; IQR, interquartile range; CI, confidence interval; MBG, mean blood glucose; NR, not reported; NS, not significant, IDeg, insulin degludec; IGlr, insulin glargine; IGla-300, Insulin glargine 300 units/mL; IGla-100, Insulin glargine 100 units/mL; IAsp, insulin aspart. * Data are presented as median (interquartile range (IQR)).

## Data Availability

Data analyzed in this study were a re-analysis of existing data, which are openly available at locations cited in the reference section.

## Supplementary Materials

The following are available online at https://www.mdpi.com/article/10.3390/metabo11090639/s1, Table S1: PRISMA check list, Table S2: Electronic search strategy, Figure S1: Funnel plots, Figure S2: Sensitivity analysis.

## References

[1] Bellido V., Suarez L., Rodriguez M.G., Sanchez C., Dieguez M., Riestra M., Casal F., Delgado E., Menendez E., Umpierrez G.E. (2015). Comparison of Basal-Bolus and Premixed Insulin Regimens in Hospitalized Patients with Type 2 Diabetes. Diabetes Care.

[2] Badlani S., Ford W.T., David J.Y., Brogan G.X., Pollack C.V., Volturo G.A.J.C.E., Reports H.M. (2014). Evidence for basal–bolus insulin versus slide scale insulin. Curr. Emerg. Hosp. Med. Rep..

[3] Wu T., Betty B., Downie M., Khanolkar M., Kilov G., Orr-Walker B., Senator G., Fulcher G. (2015). Practical Guidance on the Use of Premix Insulin Analogs in Initiating, Intensifying, or Switching Insulin Regimens in Type 2 Diabetes. Diabetes Ther..

[4] Elizarova S., Galstyan G., Wolffenbuttel B.H. (2014). Role of premixed insulin analogues in the treatment of patients with type 2 diabetes mellitus: A narrative review. J. Diabetes.

[5] Christiansen J.S., Home P., Kumar A. (2016). IDegAsp (insulin degludec + insulin aspart) for the management of type 2 diabetes: Current status. Expert Rev. Endocrinol. Metab..

[6] Haahr H., Fita E.G., Heise T. (2017). A Review of Insulin Degludec/Insulin Aspart: Pharmacokinetic and Pharmacodynamic Properties and Their Implications in Clinical Use. Clin. Pharmacokinet..

[7] Glastras S.J., Cohen N., Dover T., Kilov G., MacIsaac R.J., McGill M., Fulcher G.R. (2020). The Clinical Role of Insulin Degludec/Insulin Aspart in Type 2 Diabetes: An Empirical Perspective from Experience in Australia. J. Clin. Med..

[8] Fulcher G., Mehta R., Fita E.G., Ekelund M., Bain S.C. (2018). Efficacy and Safety of IDegAsp versus BIAsp 30, Both Twice Daily, in Elderly Patients with Type 2 Diabetes: Post Hoc Analysis of Two Phase 3 Randomized Controlled BOOST Trials. Diabetes Ther..

[9] Bode B.W., Iotova V., Kovarenko M., Laffel L.M., Rao P.V., Deenadayalan S., Ekelund M., Larsen S.F., Danne T. (2019). Efficacy and Safety of Fast-Acting Insulin Aspart Compared with Insulin Aspart, Both in Combination with Insulin Degludec, in Children and Adolescents with Type 1 Diabetes: The onset 7 Trial. Diabetes Care.

[10] Shimoda S., Sakamoto W., Hokamura A., Matsuo Y., Sekigami T., Ichimori S., Iwashita S., Ishii N., Otsu K., Yoshimura R. (2019). Comparison of the efficacy and safety of once-daily insulin degludec/insulin aspart (IDegAsp) and long-acting second-generation basal insulin (insulin degludec and insulin glargine 300 units/mL) in insulin-naïve Japanese adults with type 2 diabetes: A pilot, randomized, controlled study. Endocr. J..

[11] Semlitsch T., Engler J., Siebenhofer A., Jeitler K., Berghold A., Horvath K. (2020). (Ultra-)long-acting insulin analogues versus NPH insulin (human isophane insulin) for adults with type 2 diabetes mellitus. Cochrane Database Syst. Rev..

[12] Warren M.L., Chaykin L.B., Jabbour S., Sheikh-Ali M., Hansen C.T., Nielsen T.S., Norwood P. (2017). Insulin Degludec 200 Units/mL Is Associated with Lower Injection Frequency and Improved Patient-Reported Outcomes Compared with Insulin Glargine 100 Units/mL in Patients with Type 2 Diabetes Requiring High-Dose Insulin. Clin. Diabetes.

[13] Özçelik S., Çelik M., Vural A., Aydın B., Özçelik M., Gozu H. (2021). Outcomes of transition from premixed and intensive insulin therapies to insulin aspart/degludec co-formulation in type 2 diabetes mellitus: A real-world experience. Arch. Med. Sci..

[14] Fujimoto K., Iwakura T., Aburaya M., Matsuoka N. (2018). Twice-daily insulin degludec/insulin aspart effectively improved morning and evening glucose levels and quality of life in patients previously treated with premixed insulin: An observational study. Diabetol. Metab. Syndr..

[15] Yang W., Ma J., Hong T., Liu M., Miao H., Peng Y., Wang C., Xu X., Yang T., Nielsen A.M. (2019). Efficacy and safety of insulin degludec/insulin aspart versus biphasic insulin aspart 30 in Chinese adults with type 2 diabetes: A phase III, open-label, 2:1 randomized, treat-to-target trial. Diabetes Obes. Metab..

[16] Onishi Y., Yamada K., Zacho J., Ekelund J., Iwamoto Y. (2016). Insulin degludec/insulin aspart vs biphasic insulin aspart 30 twice daily in Japanese patients with type 2 diabetes: A randomized controlled trial. J. Diabetes Investig..

[17] Kaneko S., Chow F., Choi D.S., Taneda S., Hirao K., Park Y., Andersen T.H., Gall M.-A., Christiansen J.S. (2015). Insulin degludec/insulin aspart versus biphasic insulin aspart 30 in Asian patients with type 2 diabetes inadequately controlled on basal or pre-/self-mixed insulin: A 26-week, randomised, treat-to-target trial. Diabetes Res. Clin. Pr..

[18] Franek E., Haluzik M., Varžić S.C., Sargin M., Macura S., Zacho J., Christiansen J.S. (2015). Twice-daily insulin degludec/insulin aspart provides superior fasting plasma glucose control and a reduced rate of hypoglycaemia compared with biphasic insulin aspart 30 in insulin-naïve adults with Type 2 diabetes. Diabet. Med..

[19] Fulcher G.R., Christiansen J.S., Bantwal G., Polaszewska-Muszynska M., Mersebach H., Andersen T.H., Niskanen L.K. (2014). Comparison of Insulin Degludec/Insulin Aspart and Biphasic Insulin Aspart 30 in Uncontrolled, Insulin-Treated Type 2 Diabetes: A Phase 3a, Randomized, Treat-to-Target Trial. Diabetes Care.

[20] Niskanen L., Leiter L.A., Franek E., Weng J., Damci T., Munoz-Torres M., Donnet J.-P., Endahl L., Skjøth T.V., Vaag A. (2012). Comparison of a soluble co-formulation of insulin degludec/insulin aspart vs biphasic insulin aspart 30 in type 2 diabetes: A randomised trial. Eur. J. Endocrinol..

[21] Na Jang H., Yang Y.S., Lee S.O., Oh T.J., Koo B.K., Jung H.S. (2019). Favorable Glycemic Control with Once-Daily Insulin Degludec/Insulin Aspart after Changing from Basal Insulin in Adults with Type 2 Diabetes. Endocrinol. Metab..

[22] Cho K.Y., Nakamura A., Oba-Yamamoto C., Tsuchida K., Yanagiya S., Manda N., Kurihara Y., Aoki S., Atsumi T., Miyoshi H. (2020). Switching to Once-Daily Insulin Degludec/Insulin Aspart from Basal Insulin Improves Postprandial Glycemia in Patients with Type 2 Diabetes Mellitus: Randomized Controlled Trial. Diabetes Metab. J..

[23] Philis-Tsimikas A., Astamirova K., Gupta Y., Haggag A., Roula D., Bak B., Fita E., Nielsen A., Demir T. (2019). Similar glycaemic control with less nocturnal hypoglycaemia in a 38-week trial comparing the IDegAsp co-formulation with insulin glargine U100 and insulin aspart in basal insulin-treated subjects with type 2 diabetes mellitus. Diabetes Res. Clin. Pr..

[24] Nagai Y., Nishine A., Hashimoto E., Nakayama T., Sasaki Y., Murakami M., Ishii S., Kato H., Tanaka Y. (2017). Efficacy and safety of switching from basal insulin to once-daily insulin degludec/insulin aspart in Japanese patients with inadequately controlled type 2 diabetes: A 4-week, randomized, open-label, treat-to-target study. J. Diabetes Investig..

[25] Kumar A., Franek E., Wise J., Niemeyer M., Mersebach H., Simó R. (2016). Efficacy and Safety of Once-Daily Insulin Degludec/Insulin Aspart versus Insulin Glargine (U100) for 52 Weeks in Insulin-Naïve Patients with Type 2 Diabetes: A Randomized Controlled Trial. PLoS ONE.

[26] Kumar S., Jang H.C., Demirağ N.G., Skjøth T.V., Endahl L., Bode B. (2016). Efficacy and safety of once-daily insulin degludec/insulin aspart compared with once-daily insulin glargine in participants with Type 2 diabetes: A randomized, treat-to-target study. Diabet. Med..

[27] Onishi Y., Ono Y., Rabøl R., Endahl L., Nakamura S. (2013). Superior glycaemic control with once-daily insulin degludec/insulin aspart versus insulin glargine in Japanese adults with type 2 diabetes inadequately controlled with oral drugs: A randomized, controlled phase 3 trial. Diabetes Obes. Metab..

[28] Heise T., Tack C.J., Cuddihy R., Davidson J., Gouet D., Liebl A., Romero E., Mersebach H., Dykiel P., Jorde R. (2011). A New-Generation Ultra-Long-Acting Basal Insulin with a Bolus Boost Compared with Insulin Glargine in Insulin-Naive People with Type 2 Diabetes: A randomized, controlled trial. Diabetes Care.

[29] Kalra S., Latif Z.A., Comlekci A., Galvez G.G., Malik R., Pathan F., Kumar A. (2016). Pragmatic use of insulin degludec/insulin aspart co-formulation: A multinational consensus statement. Indian J. Endocrinol. Metab..

[30] Woo V., Berard L., Roscoe R. (2020). Understanding the Clinical Profile of Insulin Degludec, the Latest Basal Insulin Approved for Use in Canada: A Narrative Review. Diabetes Ther..

[31] Reynolds N.A., Wagstaff A.J. (2004). Insulin Aspart. Drugs.

[32] Kaneko S., da Rocha Fernandes J.D., Yamamoto Y., Langer J., Faurby M. (2021). A Japanese Study Assessing Glycemic Control with Use of IDegAsp Co-formulation in Patients with Type 2 Diabetes in Clinical Practice: The JAGUAR Study. Adv. Ther..

[33] Eledrisi M., Suleiman N.N., Salameh O., Hamad M.K., Rabadi O., Mohamed A., Al Adawi R., Salam A. (2018). Twice-daily insulin glargine for patients with uncontrolled type 2 diabetes mellitus. J. Clin. Transl. Endocrinol..

[34] Hopkinson H.E., Jacques R., Gardner K.J., Amiel S.A., Mansell P. (2015). Twice- rather than once-daily basal insulin is associated with better glycaemic control in Type 1 diabetes mellitus 12 months after skills-based structured education in insulin self-management. Diabet. Med..

[35] Nasrallah S.N., Reynolds L.R. (2012). Insulin degludec, the new generation basal insulin or just another basal insulin?. Clin. Med. Insights Endocrinol. Diabetes.

[36] Liu W., Yang X., Huang J. (2018). Efficacy and Safety of Insulin Degludec versus Insulin Glargine: A Systematic Review and Meta-Analysis of Fifteen Clinical Trials. Int. J. Endocrinol..

