# Supplementary material for: Efficacy and Safety of Insulin Degludec/Insulin Aspart Compared with a Conventional Premixed Insulin or Basal Insulin: A Meta-Analysis
Source: Metabolites. 2021 Sep 18;11(9):639. doi: 10.3390/metabo11090639 (PMC8470485; doi:10.3390/metabo11090639)

Supplementary Table S1. PRISMA check list

Section/topic	#	Checklist item	Reported on page #
**TITLE**			
Title	1	Identify the report as a systematic review, meta-analysis, or both.	1
**ABSTRACT**			
Structured summary	2	Provide a structured summary including, as applicable: background; objectives; data sources; study eligibility criteria, participants, and interventions; study appraisal and synthesis methods; results; limitations; conclusions and implications of key findings; systematic review registration number.	1
**INTRODUCTION**			
Rationale	3	Describe the rationale for the review in the context of what is already known.	1-2
Objectives	4	Provide an explicit statement of questions being addressed with reference to participants, interventions, comparisons, outcomes, and study design (PICOS).	1-2
**METHODS**			
Protocol and registration	5	Indicate if a review protocol exists, if and where it can be accessed (e.g., Web address), and, if available, provide registration information including registration number.	NA
Eligibility criteria	6	Specify study characteristics (e.g., PICOS, length of follow-up) and report characteristics (e.g., years considered, language, publication status) used as criteria for eligibility, giving rationale.	12-13
Information sources	7	Describe all information sources (e.g., databases with dates of coverage, contact with study authors to identify additional studies) in the search and date last searched.	12-13
Search	8	Present full electronic search strategy for at least one database, including any limits used, such that it could be repeated.	12-13
Study selection	9	State the process for selecting studies (i.e., screening, eligibility, included in systematic review, and, if applicable, included in the meta-analysis).	12-13
Data collection process	10	Describe method of data extraction from reports (e.g., piloted forms, independently, in duplicate) and any processes for obtaining and confirming data from investigators.	12-13
Data items	11	List and define all variables for which data were sought (e.g., PICOS, funding sources) and any assumptions and simplifications made.	12-13
Risk of bias in individual studies	12	Describe methods used for assessing risk of bias of individual studies (including specification of whether this was done at the study or outcome level), and how this information is to be used in any data synthesis.	12-13
Summary measures	13	State the principal summary measures (e.g., risk ratio, difference in means).	12-13
Synthesis of results	14	Describe the methods of handling data and combining results of studies, if done, including measures of consistency (e.g., I^2) for each meta-analysis.	12-13
Risk of bias across studies	15	Specify any assessment of risk of bias that may affect the cumulative evidence (e.g., publication bias, selective reporting within studies).	12-13
Additional analyses	16	Describe methods of additional analyses (e.g., sensitivity or subgroup analyses, meta-regression), if done, indicating which were pre-specified.	12-13
**RESULTS**			
Study selection	17	Give numbers of studies screened, assessed for eligibility, and included in the review, with reasons for exclusions at each stage, ideally with a flow diagram.	2-10, Fig 1
Study characteristics	18	For each study, present characteristics for which data were extracted (e.g., study size, PICOS, follow-up period) and provide the citations.	2, Table 1
Risk of bias within studies	19	Present data on risk of bias of each study and, if available, any outcome level assessment (see item 12).	2
Results of individual studies	20	For all outcomes considered (benefits or harms), present, for each study: (a) simple summary data for each intervention group (b) effect estimates and confidence intervals, ideally with a forest plot.	7-10 Fig 2-4
Synthesis of results	21	Present results of each meta-analysis done, including confidence intervals and measures of consistency.	7-10 Fig 2-4
Risk of bias across studies	22	Present results of any assessment of risk of bias across studies (see Item 15).	2 Fig S1
Additional analysis	23	Give results of additional analyses, if done (e.g., sensitivity or subgroup analyses, meta-regression [see Item 16]).	7-10, Fig S2,
**DISCUSSION**			
Summary of evidence	24	Summarize the main findings including the strength of evidence for each main outcome; consider their relevance to key groups (e.g., healthcare providers, users, and policy makers).	10-11
Limitations	25	Discuss limitations at study and outcome level (e.g., risk of bias), and at review-level (e.g., incomplete retrieval of identified research, reporting bias).	11
Conclusions	26	Provide a general interpretation of the results in the context of other evidence, and implications for future research.	11
**FUNDING**			
Funding	27	Describe sources of funding for the systematic review and other support (e.g., supply of data); role of funders for the systematic review.	13

Supplementary Table S2. Electronic search strategy

Database	Search terms (inception to July 10, 2021)	Number of study
PubMed<br>No filters activated	degludec and aspart	154
EMBASE<br>Filter: title, abstract or keywords	'degludec':ti,ab,kw AND 'aspart':ti,ab,kw	295
Cochrane Library<br>Filter: Trials	degludec and aspart	472

Figure S1. Funnel plots. (A) Studies with MD of FBS compared to a conventional premixed insulin, (B) Studies with MD of mean self-measured glucose level compared to a conventional premixed insulin, (C) Studies with MD of mean self-measured glucose level compared to basal insulin, (D) Studies with MD of PPG compared to basal insulin, (E) Studies with OR for nocturnal hypoglycemia compared to a conventional premixed insulin

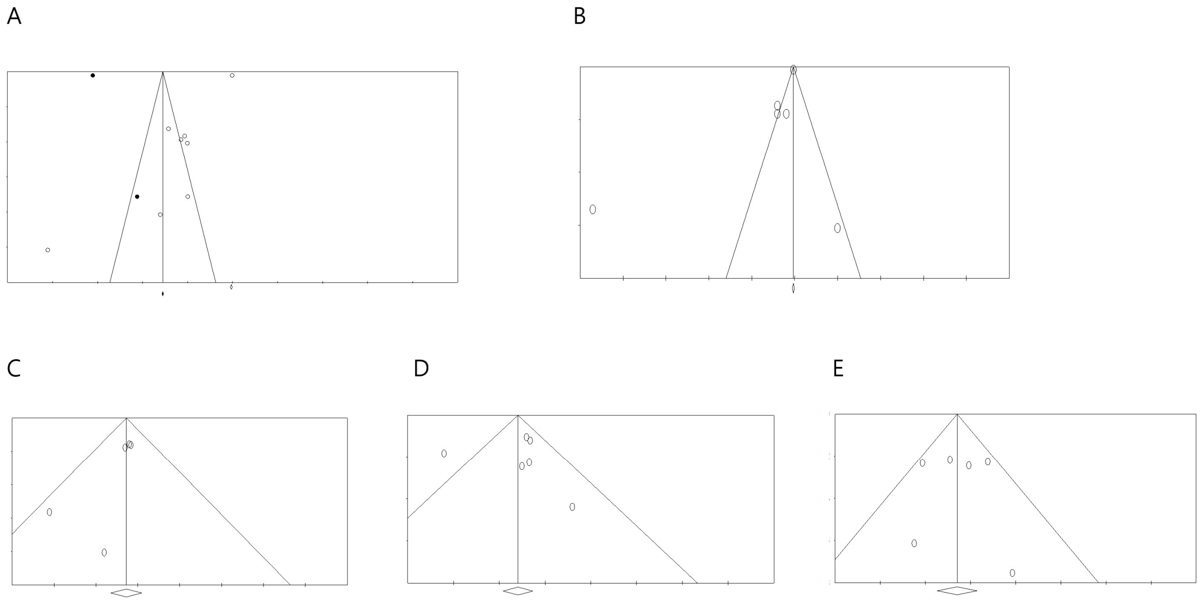

Figure S2. Sensitivity analysis. (A) Studies with MD of FBS compared to a conventional premixed insulin, (B) Studies with MD of mean self-measured glucose level compared to a conventional premixed insulin, (C) Studies with MD of mean self-measured glucose level compared to basal insulin, (D) Studies with MD of PPG compared to basal insulin, (E) Studies with OR for nocturnal hypoglycemia compared to a conventional premixed insulin

A

Study name	Point	Lower limit	Upper limit
Ozcelik et al. 2021	-1.012	-1.652	-0.371
Fujimoto et al. 2018	-1.472	-1.920	-1.024
Yang et al. 2018	-1.328	-2.057	-0.600
Onishi et al. 2017	-1.308	-2.035	-0.581
Kaneko et al. 2015	-1.392	-2.168	-0.615
Franek et al. 2015	-1.400	-2.172	-0.627
Flutcher et al. 2014	-1.378	-2.142	-0.613
Nikanen et al. 2012	-1.392	-2.137	-0.647
**Overall MD**	-1.342	-2.030	-0.654

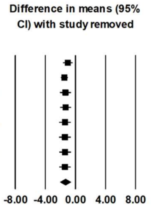

B

Study name	Point	Lower limit	Upper limit
Ozcelik et al. 2021	-0.181	-0.427	0.065
Fujimoto et al. 2018	-0.865	-1.723	-0.006
Yang et al. 2018	-0.743	-1.471	-0.015
Onishi et al. 2017	-0.822	-1.394	-0.250
Kaneko et al. 2015	-0.777	-1.467	-0.087
Flutcher et al. 2014	-0.724	-1.401	-0.046
**Overall MD**	-0.635	-1.176	-0.093

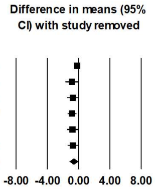

C

Study name	Point	Lower limit	Upper limit
Tsimikas et al. 2019	-0.658	-0.886	-0.429
Cho et al. 2019	-0.634	-0.820	-0.448
Nagai et al. 2017	-0.612	-0.799	-0.425
Kumar et al. 2016 a	-0.633	-0.850	-0.416
Kumar et al. 2016 b	-0.666	-0.892	-0.441
**Overall MD**	-0.638	-0.822	-0.453

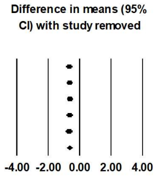

D

Study name	Point	Lower limit	Upper limit
Cho et al. 2019 (morning injection)	-1.734	-2.399	-1.069
Cho et al. 2019 (evening injection)	-1.663	-2.408	-0.918
Kumar et al. 2016 a	-1.715	-2.566	-0.864
Kumar et al. 2016 b	-1.691	-2.575	-0.808
Ornishi et al. 2013	-1.349	-1.698	-1.000
Heise et al. 2011	-1.694	-2.445	-0.944
**Overall MD**	-1.644	-2.282	-1.006

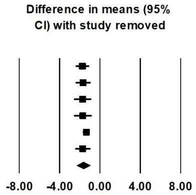

E

Study name	Statistics with study removed				
	Point	Lower limit	Upper limit	Z-Value	p-Value
Onishi et al. 2021	0.515	0.412	0.644	-5.836	0.000
Yang et al. 2018	0.505	0.394	0.646	-5.416	0.000
Kaneko et al. 2015	0.468	0.364	0.603	-5.877	0.000
Franek et al. 2015	0.586	0.456	0.753	-4.176	0.000
Flutcher et al. 2014	0.537	0.415	0.694	-4.750	0.000
Nikanen et al. 2012	0.531	0.425	0.664	-5.538	0.000
**Overall OR**	0.523	0.419	0.651	-5.778	0.000

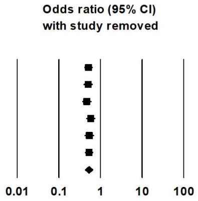

Supplement: Supplementary file 1 [file metabolites-11-00639-s001.zip › metabolites-1352952-supplementary.pdf]
